# RNAi-mediated silencing of a pyruvate dehydrogenase kinase enhances triacylglycerol biosynthesis in the oleaginous marine alga *Nannochloropsis salina*

**DOI:** 10.1038/s41598-017-11932-4

**Published:** 2017-09-13

**Authors:** Xiaonian Ma, Lina Yao, Bo Yang, Yuan Kun Lee, Feng Chen, Jin Liu

**Affiliations:** 1 Institute for Food and Bioresource Engineering, College of Engineering, Peking University, Beijing, 100871 China; 2 BIC-ESAT, College of Engineering, Peking University, Beijing, 100871 China; 3 Department of Microbiology and Immunology, Yong Loo Lin School of Medicine, National University of Singapore, 117545 Singapore, Singapore

## Abstract

Oleaginous microalgae have been emerging as the third-generation feedstocks for biofuel production. Genetic manipulation for improving triacylglycerol (TAG) accumulation represents a promising approach towards the economics of microalgal biofuels. Acetyl-CoA, the essential carbon precursor for *de novo* fatty acid biosynthesis, can be derived from pyruvate catalyzed by pyruvate dehydrogenase, which is negatively regulated by pyruvate dehydrogenase kinase (PDK). In the present study, we characterized a *PDK* gene (*NsPDK*) from *Nannochloropsis salina*. Subcellular localization assay assisted by green fluorescence protein (GFP) fusion indicated the localization of *NsPDK* in mitochondria of *N. salina* cells. *NsPDK* knockdown via RNA interference strategy attenuated *NsPDK* expression at the mRNA level and its enzymatic activity *in vivo*, leading to faster TAG accumulation without compromising cell growth under high light stress conditions. Interestingly, the TAG increase was accompanied by a decline in membrane polar lipids. *NsPDK* knockdown also altered fatty acid profile in *N. salina*. Furthermore, transcriptional analysis suggested that the carbon metabolic pathways might be influenced by *NsPDK* knockdown leading to diverted carbon flux towards TAG synthesis. Taken together, our results demonstrate the role of *NsPDK* in regulating TAG accumulation and provide valuable insights into future manipulation of oleaginous microalgae for improving biofuel production.

## Introduction

The increasing concerns about the shortage of fossil fuels and the burning-associated environmental problems have led to interest in developing sustainable and renewable biofuels in recent years^1^. Microalgae are considered as a promising feedstock for biofuels, in that they grow fast with high photosynthetic efficiency, have no competition with crops for arable lands, and can accumulate large quantities of triacylglycerols (TAGs), an ideal precursor for making biodiesel via transesterification^2, 3^. Although many advances have been achieved for microalgal biofuels, challenges remain to be addressed to bring down the production cost for commercial uses^4^. Efforts are being made to improve lipids, TAGs in particular, by rational genetic engineering based on the understanding of regulatory mechanisms involved in lipid metabolism^5, 6^.

TAG biosynthesis is believed to be mediated mainly via two pathways, acyl-CoA independent pathway and acyl-CoA dependent Kennedy pathway^7^. The latter pathway contributes largely to the TAG accumulation under abiotic stress conditions, which starts from glyceol-3-phosphate with three sequential acylation steps^8^. The acyls in TAG can be either from recycled membrane lipids or from *de novo* synthesized fatty acids (FAs), both with the primary precursor being acetyl-CoA. Li *et al*.^9^ found out that most of the genes involved in *de novo* FA synthesis were downregulated while total fatty acid accumulation was significantly enhanced in *Nannochloropsis* under nitrogen starvation. This could explain why previous attempts to increase lipid content by overexpressing acetyl-coA carboxylase or fatty acid synthase had limited success^10–12^. It has been speculated that provision of acetyl-CoA may be the rate-limiting step for TAG biosynthesis in *Nannochloropsis*. Pyruvate dehydrogenase complex (PDHC) is a multienzyme complex catalyzing the oxidative decarboxylation of pyruvate to yield acetyl-CoA and NAD(P)H^13^. PDHC contains three primary components: pyruvate dehydrogenase (E1), dihydrolipoyl acetyltransferase (E2) and dihydrolipoyl dehydrogenase (E3). Higher plants contain two distinct isoforms of PDHC, one located in mitochondria and the other in the plastid stroma^14^. Mitochondrial PDHC becomes inactive once Ser-phosphorylation occurs in the α-subunit of PDHC E1 component, which is catalyzed by pyruvate dehydrogenase kinase (PDK)^13, 14^. The characterization of PDK has been conducted in some higher plants as well as microalgae^14–16^. Overexpression of a *PDK* gene from *Brassica napus* in Arabidopsis resulted in a decrease in both the PDHC activity and seed oil content^14^. *PDK* knockdown, on the other hand, made Arabidopsis accumulate elevated oil levels in seeds^17^. Recently, it was reported that the antisense knockdown of *PDK* promoted neutral lipid accumulation in the diatom *Phaeodactylum tricornutum*, but with compromised cell growth^15^. Besides, only neutral lipids were analyzed based on Nile red staining in this study; the effects of *PDK* knockdown on TAG and polar lipids content remained unknown.

*Nannochloropsis* has been emerging as an oleaginous model alga to study TAG biosynthesis and regulation because of its fast growth, high TAG content, available genome sequence, and established genetic tools^9, 18, 19^. *Nannochloropsis salina* has been reported to be successfully transformed^18^. Here we report the characterization of a *PDK* gene (*NsPDK*) from *Nannochloropsis salina*. Assisted with the enhanced green fluorescence protein fusion, NsPDK was found to be positioned likely in mitochondria. *NsPDK* knockdown via RNA interference strategy not only promoted total lipid content and altered fatty acid profiles, but also enhanced TAG accumulation, while without slowing down cell growth. Transcriptional analysis suggested the possible gene regulatory network of PDK and its knockdown may bring more carbon precursors and reducing powers for *de novo* fatty acid synthesis thereby enhancing TAG accumulation. Our work helps understand the role of PDK in regulating carbons for lipid biosynthesis and provides insights into target genetic engineering of *Nannochloropsis* for trait improvement and production uses.

## Results

### Sequence analysis of *NsPDK*

**Figure 1:**
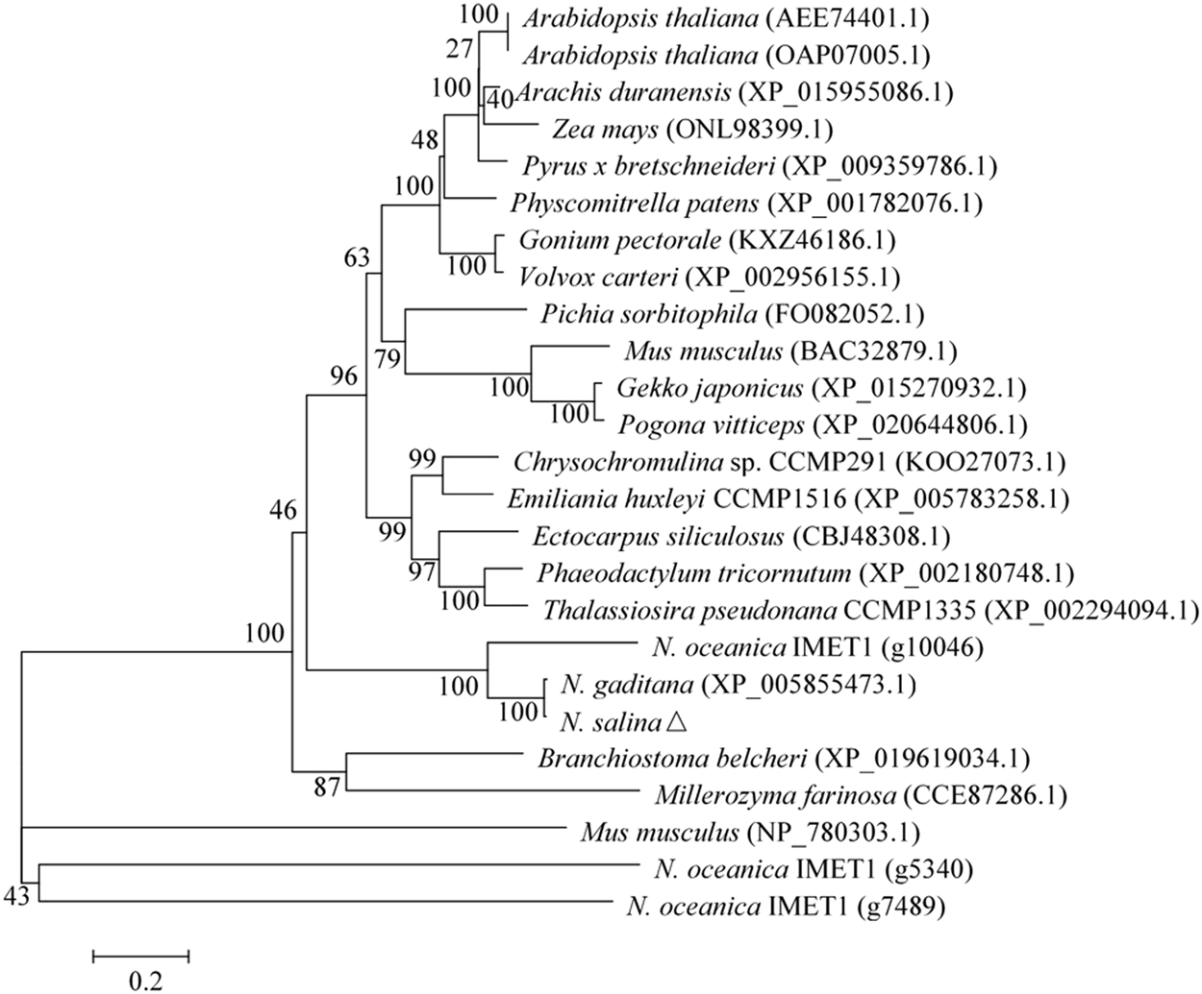
Phylogenetic tree based on protein sequence of PDK from *N. salina*. The GenBank accession number or gene ID of each sequence was shown in the parenthesis. The tree was constructed using the neighbor-joining method (1000 bootstraps). The scale bar (0.2) was shown in the unit of base substitutions per site.

It has been reported that the genome of *N. salina* are highly identical to *N. gaditana* ^19–21^. Using the *PDK* sequence from *N. gaditana* as the reference, we successfully amplified *NsPDK* sequence (Supplementary Data 1). The phylogenetic analysis based on amino acid sequences revealed that the putative NsPDK was closely clustered with PDK from *N. gaditana* and *N. oceanica* IMET1 (Fig. 1). By analyzing with the software TargetP 1.1 (http://www.cbs.dtu.dk/services/TargetP/), NsPDK was predicted to have a transit peptide targeting to mitochondrion.

### *In vivo* localization of NsPDK

**Figure 2:**
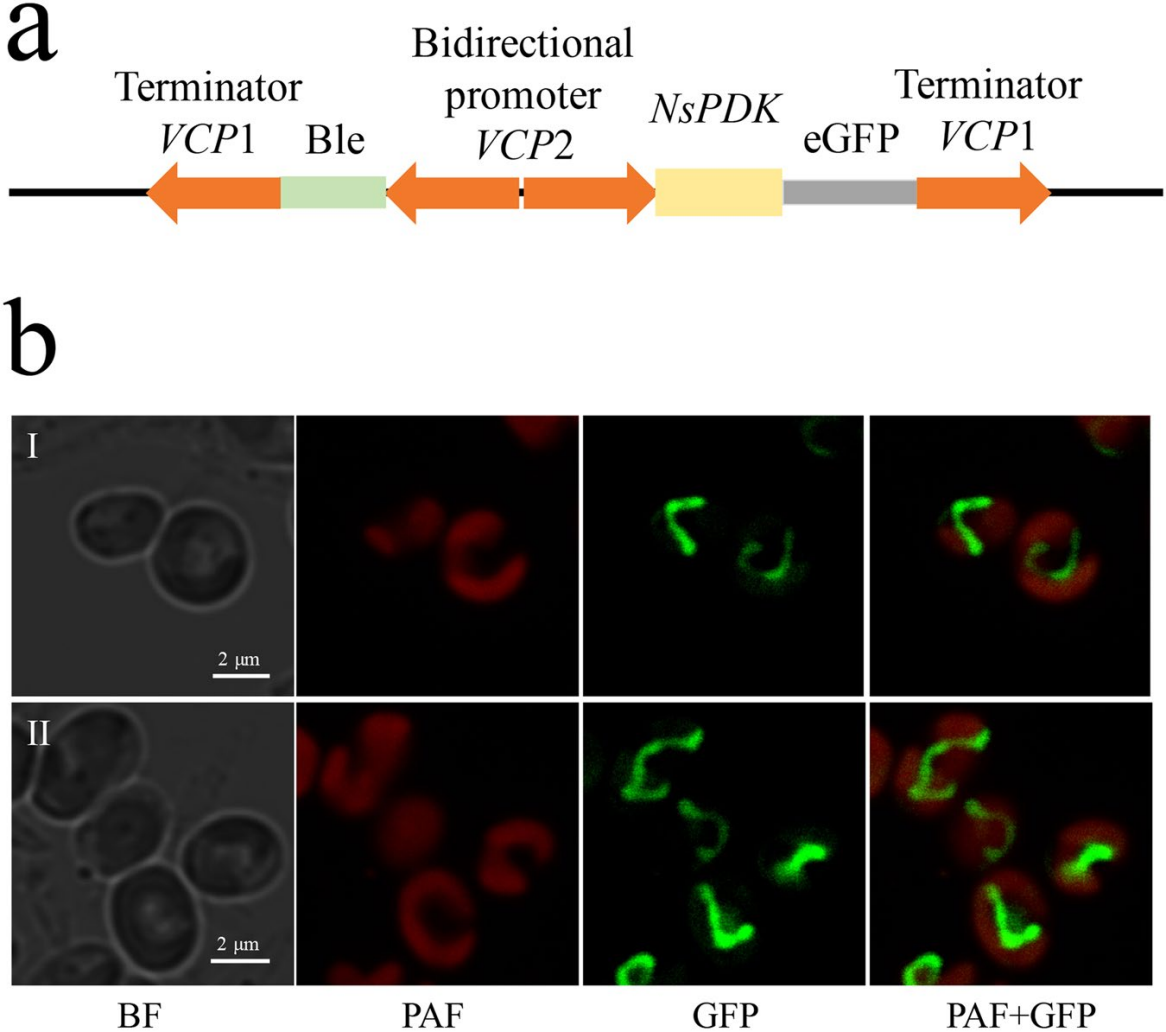
Subcellular localization of NsPDK in *N. salina* cells. (**a**) The construction of pPha-VCP-*Ns*PDK-eGFP vector. (**b**) Microscopy images of two representative transgenic lines (I and II) expressing NsPDK-GFP fusion. From left to right: BF (bright field), PAF (plastid autofluorescence), GFP (green fluorescent protein), and PAF + GFP, merge of PAF and GFP.

In order to examine the subcellular localization of NsPDK, we constructed the NsPDK-GFP fusion (Fig. 2a) and had it introduced into *N. salina*. With a confocal microscope, it was observed that the GFP fluorescence appeared as tubular structures next to, but not merged with the plastid, as indicated by the plastid autofluorescence (PAF) in red (Fig. 2b). By comparing a recent subcellular localization analysis in *N. oceanica* ^22^, NsPDK is likely positioned in mitochondria. These results further proved that NsPDK functioned as the regulator of mitochondrial PDHC.

### Genotypical characterization of *NsPDK* knockdown algal lines

**Figure 3:**
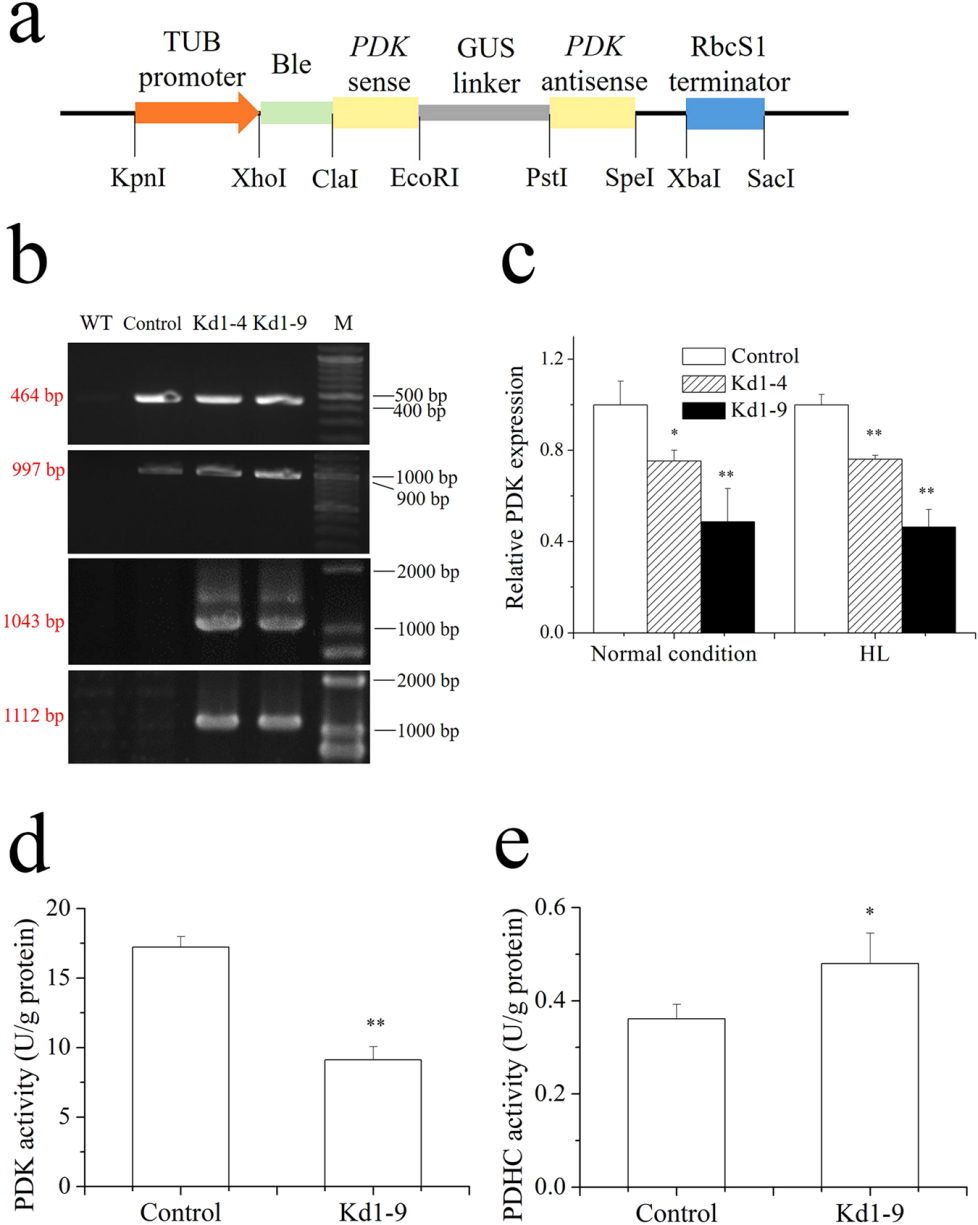
The RNAi vector and identification of plasmids in transgenic cells. (**a**) Schematic diagram of plasmid pGreen II 0000. (**b**) Detection of bleomycin resistant fragment (464 bp), GUS region (997 bp), fragment crossing sense and GUS sequence (1043 bp) and fragment crossing GUS and antisense sequence (1112 bp) from transgenic *N. salina* (shown in four cropped panels, and full-length gels are presented in Supplementary Fig. 1). (**c**) Transcriptional abundance of *PDK* in transformants determined by qRT-PCR. The data was obtained on day 6 with normal condition and day 2 with the stress of high light. The endogenous actin gene was used as an internal standard and the mRNA abundance of control was set as 1. (**d**) PDK activity of Kd1-9 and control. (**e**) PDHC activity of Kd1-9 and control. The data was obtained on day 2 with the stress of high light for d and e. TUB, β-tubulin; PDK, pyruvate dehydrogenase kinase; GUS, β-glucuronidase; RbcS1, ribulose-1,5-bisphosphate carboxylase/oxygenase small subunit. WT, wild type; Control, empty vector introduced *N. salina*; Kd1-4 and Kd1-9, transgenic *N. salina*; M, DNA ladder marker. Values are presented with average and SD from at least three biological replicates. Asterisk (*p < 0.05 and **p < 0.01) means significant differences between control and transformants, using independent samples t-test and one way ANOVA Turkeys HSD test.

The RNAi vector (Fig. 3a) was introduced into *N. salina* cells via electrotransformation. The zeocin-resistant colonies were selected and cultivated as putative transgenic lines. Genomic PCR was used to confirm the integration of the vector into the genome of transgenic lines. With the primers from both GUS linker and bleomycin coding region, the corresponding 464 bp and 997 bp PCR products were detected for *NsPDK* transgenic lines (Kd1-4 and Kd1-9) and the control line transformed with empty vector, but not in wild type. By contrast, with the primers crossing *PDK* and *GUS* sequence, the expected 1043 bp and 1112 bp bands were amplified from transgenic lines instead of wild type and control line (Fig. 3b). This proved the successful transformation of *N. salina*. The stress of high light (HL) was applied to induce lipid accumulation in transgenic *N. salina*. Two-stage cultivation strategy was employed with normal illumination (50 μmol/m^2^·s) in the first stage followed by HL condition (250 μmol/m^2^·s) in the second stage. *N. salina* showed an impaired cell growth with HL as compared to normal condition (Supplementary Fig. 2), indicating HL used in this study could be considered as stress condition. In order to evaluate the knockdown efficiency, quantitative real-time PCR was conducted. The results suggested that Kd1-4 and Kd1-9 exhibited a significant decrease in *NsPDK* expression under normal condition (by 25.6% and 51.3%, respectively) and HL condition (by 23.8% and 53.6%, respectively) as compared to the empty vector control line (Fig. 3c). The transgenic line Kd1-9 with a stronger knockdown efficiency was selected for further investigations. Kd1-9 showed a 53% decrease in NsPDK activity and a 33% increase in PDHC activity (Fig. 3d and e).

### Growth, pigment and photosynthetic parameters of transgenic *N. salina*

**Figure 4:**
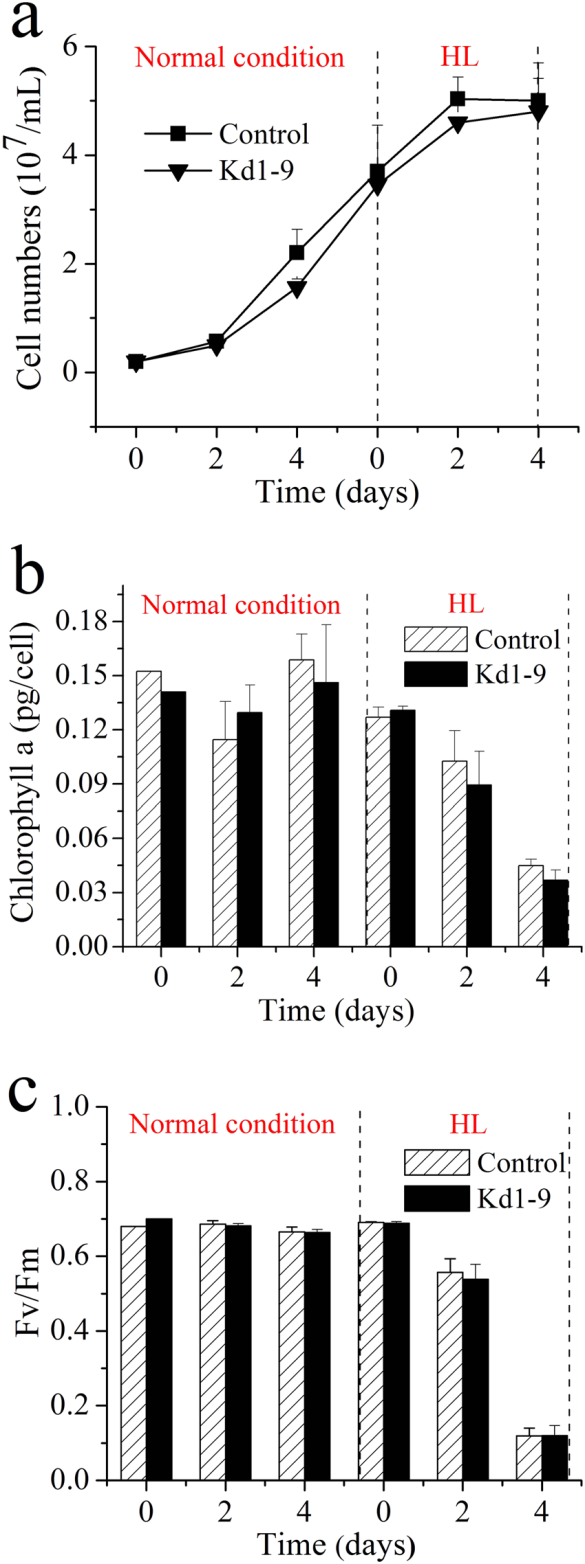
Time curve of cell numbers (**a**), chlorophyll a content (**b**) and photosynthetic parameter Fv/Fm (**c**) of transgenic cells. Control, empty vector transgenic line. Kd1-9, *PDK* knockdown line. Two stage cultivation was applied with normal cultivation (50 μmol/m^2^·s) in the first stage followed by HL (250 μmol/m^2^·s) induced lipid accumulation in the second stage. Values are presented with average and SD from at least three biological replicates, and independent samples t-test was used to determine the significance.

We examined the growth and photosynthetic parameters of transgenic *N. salina* lines. No significant difference in the cell density, chlorophyll a content or Fv/Fm was observed between Kd1-9 and empty vector control (Fig. 4), indicating that *NsPDK* knockdown had little effect on either growth or photosynthetic efficiency in *N. salina*.

### The effect of *NsPDK* knockdown on lipids, protein and carbohydrate

**Figure 5:**
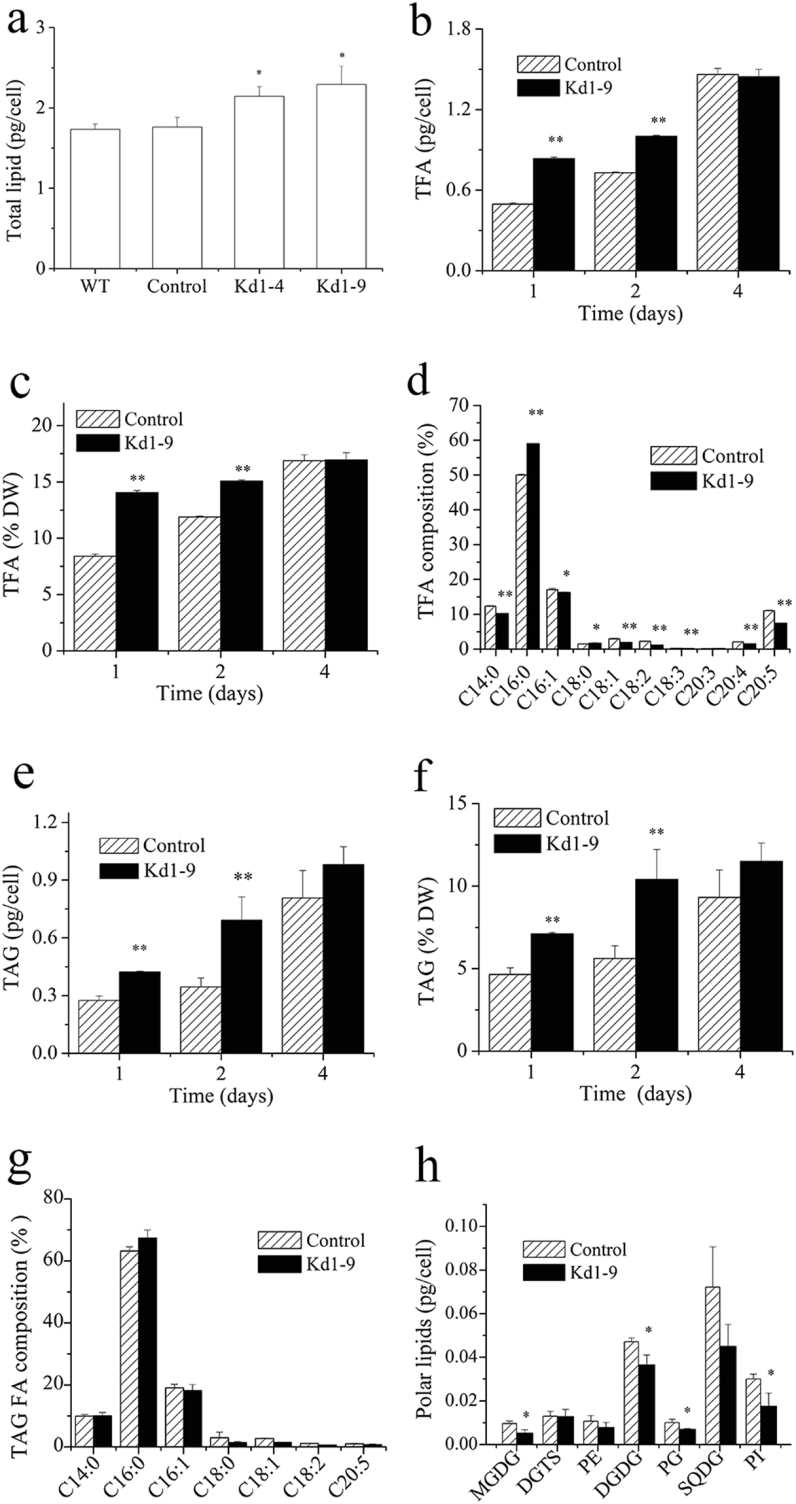
Lipids accumulation in *PDK* RNAi line Kd1-9 and empty vector introduced control line. (**a**) Total lipid content in wild type, control and Kd1-9 cells on day 2 under HL conditions. (**b**) and (**c**) Total fatty acid content in control and Kd1-9 cells on day 1, 2 and 4 under HL conditions. (**d**) Fatty acid profile in control and Kd1-9 cells on day 2 under HL conditions. (**e**) and (**f**) TAG content in control and Kd1-9 cells on day 1, 2 and 4 under HL conditions. (**g**) TAG-derived fatty acid profile in control and Kd1-9 cells on day 2 under HL conditions. (**h**) Polar lipid content in control and Kd1-9 cells on day 2 under HL conditions. Values are presented with average and SD from at least three biological replicates. Asterisk (*p < 0.05 and **p < 0.01) means significant differences between control and transformant, using independent samples t-test.

Although *PDK* knockdown showed no effect on lipid accumulation when cells grew under normal illumination conditions (Supplementary Fig. 3), it affected lipid synthesis significantly when cells were cultured under HL stress conditions. For example, the lipid accumulation was enhanced in Kd1-4 and Kd1-9 on day 2 (by 21.6 and 30.1%, respectively) as compared to the control (Fig. 5a). Accordingly, the total fatty acid (TFA) synthesis was significantly enhanced in Kd1-9 during the first two days (Fig. 5b and c). We further studied the effect of *NsPDK* knockdown on total fatty acid profile (Fig. 5d). C14:0, C16:0, C16:1 and C20:5 were the main fatty acids and together accounted for about 80% of total fatty acids in both the control and Kd1-9. The fatty acid profile was altered following the downregulated *PDK* expression, with an increase in the relative content of C16:0 and a decline in C14:0 and polyunsaturated fatty acids (C18:2, C18:3, C20:4 and C20:5). The knockdown of *NsPDK* promoted the TAG accumulation by 100% (per cell) and 86% (per dry weight) in Kd1-9 on day 2 (Fig. 5e and f). However, TAG content showed no difference between Kd1-9 and control on day 4. C16:0 was the major fatty acid in TAG derived fatty acids, followed by C16:1 and C14:0. But *NsPDK* knockdown had little effect on the fatty acid profile of TAG (Fig. 5g). Noteworthily, *NsPDK* knockdown led to differential changes in in membrane polar lipids: a considerable decrease in monogalactosyl diacylglycerol (MGDG), digalactosyl diacylglycerol (DGDG), phosphatidylglycerol (PG) and phophatidylinositol (PI), while no change in diacylglycerol-N,N,N-trimethylhomoserine (DGTS), phosphatidylethanolamine (PE) and sulfoquinovosyldiacylglycerol (SQDG), (Fig. 5h). Interestingly, NsPDK knockdown showed little effect on lipid content under nitrogen deficiency conditions (Supplementary Fig. 4), a commonly used stress for inducing lipid accumulation in microalgae^18^.

**Figure 6:**
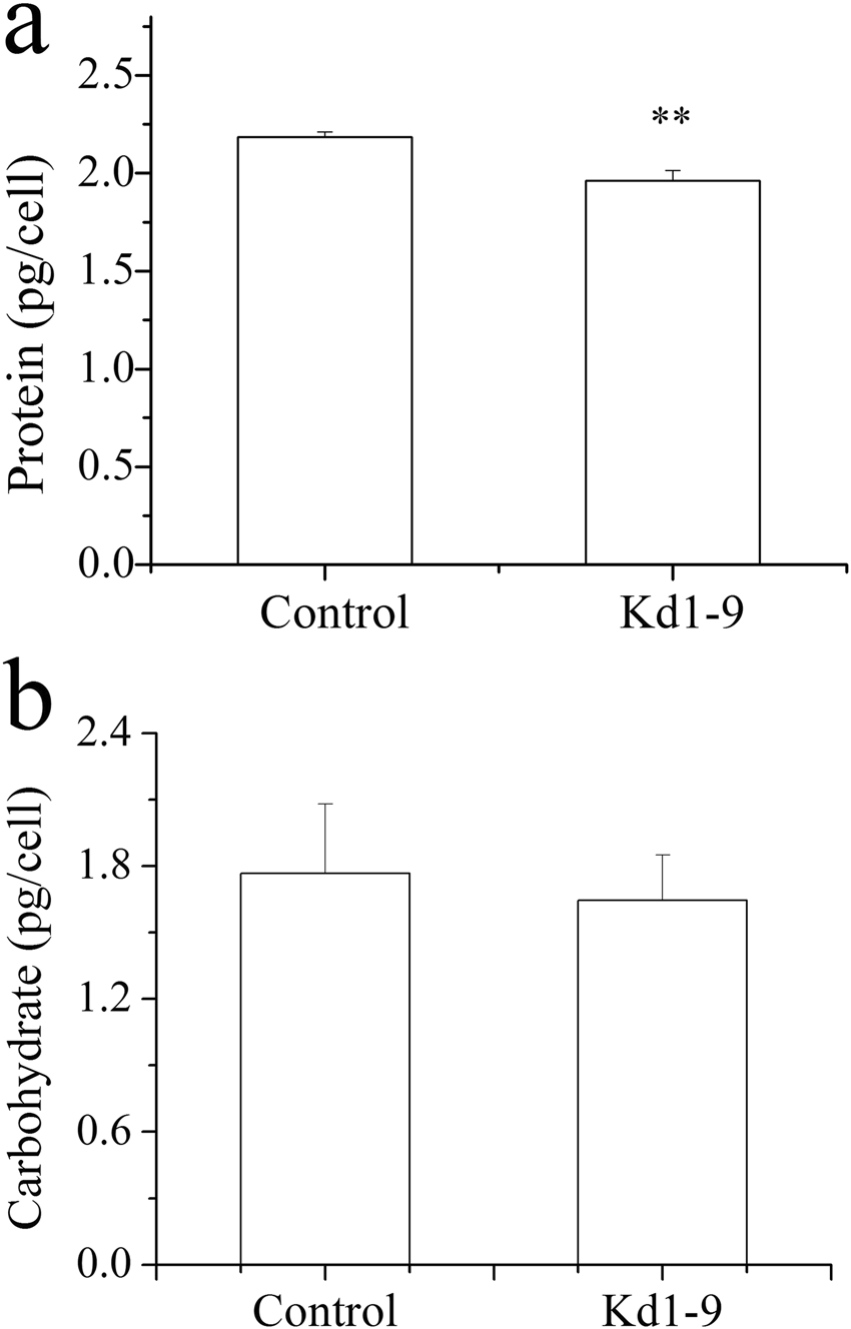
Total protein (**a**) and carbohydrate (**b**) accumulation in *NsPDK* knockdown line Kd1-9 and empty vector introduced control line on day 2 under HL conditions. Values are presented with average and SD from at least three biological replicates. Asterisk (**p < 0.01) means significant differences between control and transformant, using independent samples t-test.

Protein and carbohydrate are the major macromolecules synthesized in microalgal cells. The total protein content decreased slightly in Kd1-9, which was 10% lower than control (Fig. 6a). The carbohydrate content, on the other hand, was not significantly affected by *NsPDK* knockdown (Fig. 6b).

### Transcriptional regulation of key genes in transgenic *N. salina*

**Figure 7:**
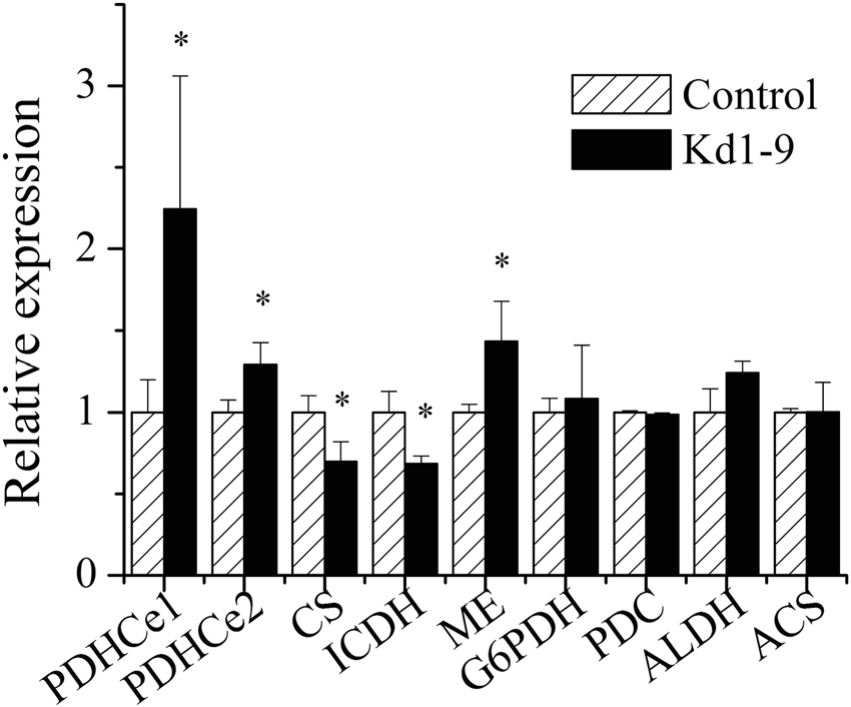
Relative expression levels of key genes in transgenic cells after 42 hours of cultivation with high light. The mRNA abundance of control was set as 1. Values are presented with average and SD from at least three biological replicates. Asterisk (*p < 0.05) means significant differences between control and Kd1-9, using independent samples t-test. Control, empty vector transgenic line; Kd1-9, *NsPDK* knockdown line. *PDHC*e1, pyruvate dehydrogenase complex E1 component; *PDHC*e2, pyruvate dehydrogenase complex E2 component; *CS*, citrate synthase; *ICDH*, isocitrate dehydrogenase; *ME*, malic enzyme; *G6PDH*, glucose-6-phosphate dehydrogenase; *PDC*, pyruvate decarboxylase; *ALDH*, aldehyde dehydrogenase; *ACS*, acetyl-CoA synthase.

The transcript abundance of key genes was analyzed by qPCR (Fig. 7). *NsPDK* knockdown led to elevated *PDHC* E1 component (*PDHC*e1) expression level (~2.2 fold). The mRNA abundance of *PDHC* E2 component (*PDHC*e2) in transgenic line was upregulated following *NsPDK* knockdown. The TCA cycle involved key genes: citrate synthase (*CS*) and isocitrate dehydrogenase (*ICDH*) were downregulated in Kd1-9 by 30% and 31%, respectively. Malic enzyme (*ME*), catalyzing the provision of NADPH as reductant for *de novo* fatty acid synthesis^23^, was upregulated. Glucose-6-phosphate dehydrogenase (*G6PDH*), involved in pentose phosphate pathway, is considered to be an ancillary means of producing NADPH in oleaginous microorganisms^23^. However, *G6PDH* was not influenced in *N. salina* by the *NsPDK* knockdown. Pyruvate can also be converted to acetate by a pyruvate decarboxylase (PDC) and an aldehyde dehydrogenase (ALDH), which is called “PDHC bypass” to produce acetyl-CoA^9^. There was no change in either *PDC* or *ALDH* expression level. The transcriptional abundance of acetyl-CoA synthase (*ACS*) was not influenced by *NsPDK* knockdown.

## Discussion

*Nannochloropsis* represents a genus of marine microalgae with high photosynthetic efficiency and can convert carbon dioxide to storage lipids mainly in the form of TAGs^24^. It has been reported that total lipid accumulation in *Nannochloropsis* can be largely enhanced (up to 60% dry weight) by stress conditions, such as nitrogen deficiency and excess light, indicative of the potential of this alga for biofuels applications^18, 25, 26^. The reported successful genetic manipulation of *Nannochloropsis* ^20, 27–29^ points to new directions for trait improvement.

The previous report on transcriptomes in *Nannochloropsis* has indicated that the key enzymes involved in *de novo* FA biosynthesis may be present in excess, and the provision of carbon precursor could be more crucial for FA production^9^. Acetyl-CoA is the vital carbon precursor for *de novo* fatty acid synthesis. PDHC catalyzes the generation of acetyl-CoA, and the activity is primarily regulated by phosphorylation by PDK. Consequently, PDK is considered to play a key role in the regulation of FA biosynthesis. The genetic manipulation of *PDK* in higher plants and microalga has suggested the vital role of PDK in lipid accumulation^14, 15^. Here we engineered a *N. salina* strain with lowered *NsPDK* expression level, with the aim to channel carbon precursors into fatty acid synthesis.

The predicted NsPDK in the present study is likely localized in the mitochondria (Fig. 2), in line with the previous studies in *P. tricornutum* and higher plants^15, 16^. The mitochondrial PDHC expression is responsive to light^13^, and the stress of high light can stimulate TAG accumulation in *Nannochloropsis species* ^24, 30^. In the present study, both the transcriptional abundance and the enzymatic activity of PDK were lowered in the transgenic line Kd1-9 as compared to control under HL stress conditions (Fig. 3c and d). The knockdown efficiency in the present study was similar to the reported investigations in *P. tricornutum* ^15^. In line with the previous reports^24, 31^, HL led to decreased chlorophyll content and Fv/Fm. Unlike in *P. tricornutum* ^15^, NsPDK knockdown showed little effect on cell growth and photosynthetic activity in *N. salina* (Fig. 4).

Enhanced total lipid content and slightly decreased protein content in transgenic cells might be due to the carbon reallocation between lipid and protein components (Figs 5a and 6a). *NsPDK* knockdown significantly accelerated the biosynthesis of TFA and TAG in *N. salina* in the first two days with high light (Fig. 5b, c, e and f). The doubled TAG content was accompanied by a considerable decrease in membrane lipid content (Fig. 5e and h), which may be due to the involvement of membrane lipid turnover for TAG assembly. The altered fatty acid profile was observed in transgenic line with a higher C16:0 proportion and a reduced C20:5 proportion (Fig. 5d). This might be caused by augmented TAG synthesis in Kd1-9 because TAG had a considerably higher percentage of saturated/monounsaturated fatty acids and a lower proportion of polyunsaturated fatty acids (Fig. 5g). It was different in the diatom *P. tricornutum* that antisense knockdown of *PDK* did not have much impact on fatty acid profile^15^. These biochemical results altogether indicated that TAG biosynthesis was promoted by *NsPDK* knockdown in *N. salina* without compromising cell growth.

**Figure 8:**
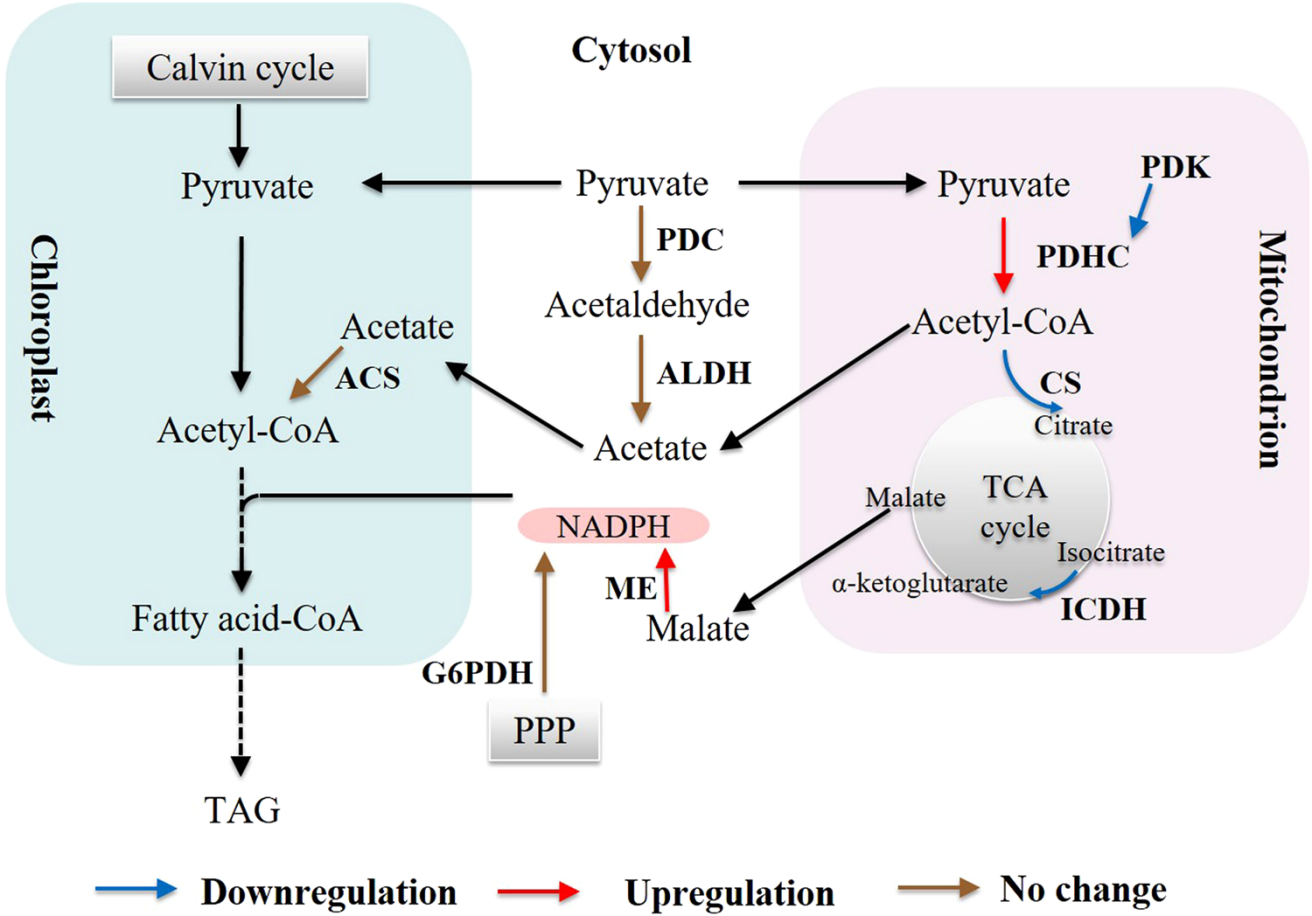
Transcriptional regulation of key genes in transgenic *N. salina*. Transcriptional regulation of genes is indicated by brown (not changed), blue (downregulated) and red (upregulated) arrows. *PDK*, pyruvate dehydrogenase kinase; *PDHC*, pyruvate dehydrogenase complex; *CS*, citrate synthase; *ICDH*, isocitrate dehydrogenase; *ME*, malic enzyme; *G6PDH*, glucose-6-phosphate dehydrogenase; *PDC*, pyruvate decarboxylase; *ALDH*, aldehyde dehydrogenase; *ACS*, acetyl-CoA synthase; PPP, pentose phosphate pathway; TAG, triacylglycerol.

The proposed working model for TAG accumulation induced by *NsPDK* knockdown in *N. salina* was depicted in Fig. 8. *PDK* attenuation could result in the activation of PDHC enzyme, and might alter the regulation of *PDHC* expression. An expected higher PDHC activity may be attributed to a lower phosphorylation status of PDHCe1 following *NsPDK* knockdown and the marked elevation of mitochondrial *PDHC* expression. Consequently, an increase in the production of acetyl-CoA in *NsPDK* knockdown line may occur, which can be channeled to chloroplast for *de novo* fatty acid synthesis^25, 32–34^. *Nannochloropsis* tends to produce large amounts of TAGs in response to high light stress^3^. ME may be activated and produces more NADPH to support the enhanced *de novo* fatty acid synthesis, therefore contributing to elevated TAG accumulation. More malate may be catalyzed to provide NADPH and less malate participates in TCA cycle in mitochondria, which attenuates TCA cycle in *NsPDK* knockdown line. This hypothesis was partly supported by the downregulated expression of *CS* and *ICDH*. It has been reported that the suppression of *CS* via RNAi affected the carbon flux channeling and increased the TAG content in *Chlamydomonas reinhardtii* ^35^. However, we didn’t observe the expected upregulation of “PDHC bypass” pathway, which may be explained by the involvement of post-transcriptional regulation. Taken all together, *NsPDK* knockdown in *N. salina* is likely to divert more carbon flux towards TAG synthesis and activate NADPH supplying pathway, leading to an overall enhanced TAG accumulation.

Our present study demonstrated that RNAi knockdown of *NsPDK* significantly promoted TAG accumulation in *N. salina*. The transgenic cells exhibited similar growth, reduced *NsPDK* expression and inhibited PDK activity as compared to empty vector control. Consequently, both the total fatty acid and TAG content were significantly enhanced following *NsPDK* knockdown, accompanied by an obvious decline in membrane lipids. The fatty acid composition was also altered in knockdown mutant. The transcriptional analysis of key genes involved in fatty acid synthesis indicated the channeling of carbon flux into TAG synthesis may occur. These attempts provide sights into genetic manipulation of microalgae as feedstocks for biofuel production.

## Methods

### Microalga and culture conditions

*N. salina* (CCMP 537) was obtained from the National Center for Marine Algae and Microbiota (NCMA, USA). The medium used in this study was modified f/2 medium (Guillard and Ryther, 1962) which contains (per liter): 375 mg NaNO_3_, 5.65 mg NaH_2_PO_4_·2H_2_O, 3.15 mg FeCl_3_·6H_2_O, 4.36 mg Na_2_EDTA·2H_2_O, 0.01 mg CoCl_2_·6H_2_O, 0.18 mg MnCl_2_·4H_2_O, 22 μg ZnSO_4_·7H_2_O, 6.3 μg Na_2_MoO_4_·2H_2_O, 9.8 μg CuSO_4_·5H_2_O and 20 g sea salts (Sigma-Aldrich, USA). The exponentially growing cells was inoculated at a cell density of 2 × 10^6^/mL (Day 0) into 250-mL flasks containing 100 mL medium. Cells cultivated with normal illumination (50 μmol/m^2^·s) for 6 days were collected (by centrifugation at 5,000 rpm for 10 min) and then resuspended in the fresh medium illuminated with a light intensity of 250 μmol/m^2^·s (high light, HL). The experiments were conducted at 25 ± 1 °C with continuous shaking at 110 rpm in a thermostatic shaker.

### Cloning and sequence prediction of PDK

The putative *N. salina PDK* sequence (*NsPDK*) was amplified based on the *PDK* gene of *N. gaditana* (Genbank accession number XM_005855411). *N. salina* cells were harvested by centrifugation at 5,000 rpm for 10 min, and the total RNA was extracted with a RNeasy Plant Mini Kit (QIAGEN, Germany) according to the manufacturer’s instructions. The reverse transcription was performed with SuperScript III First-strand Synthesis System (Invitrogen, USA) for cDNA synthesis. All the primers were shown in Supplementary Table 1. Amino acid sequence alignment and similarity among species were determined by MEGA6 using sequences from NCBI (http://blast.ncbi.nlm.nih.gov/Blast.cgi) and QIBEBT EnergyAlgae DB (http://www.bioenergychina.org:8989/). The phylogenetic tree of protein clusters was constructed by MEGA6 using the neighbor-joining method.

### Subcellular protein localization of NsPDK

The pPha-VCP vector containing the bidirectional *VCP2* promoter (from *N. oceanica*) and *VCP1* 3’UTR terminator (from *N. oceanica*) as well as *sh ble* resistance gene was constructed^22, 36^. The enhanced green fluorescent protein (eGFP) fragment was amplified by PCR from the pPhaT1-eGFP vector^36^ using the primers eGFP_ KpnI_f and eGFP_XbaI_r and ligated into the pPha-VCP vector (between the KpnI and XbaI sites), and was named as pPha-VCP-eGFP. For the localization analysis of PDK, a construct was generated to express C-terminal GFP fusion proteins in *N. salina*. The ORF of PDK was amplified by PCR from the cDNA using the primers PDK_KpnI_f and PDK_KpnI_r. The PCR products were inserted into the KpnI sites, which reside immediately upstream of the e*GFP* sequence within the pPha-VCP-eGFP vector. The vector was introduced into wild-type *N. salina* by electroporation using Bio-Rad GenePulser Xcell apparatus (Bio-Rad, USA) at 12,000 V/cm, 50 µFD and 600 Ω. Zeocin (Invitrogen, USA) at the final concentration of 2 μg/ml (agar medium) and 1 μg/ml (liquid medium) was used for selecting transgenic cells. GFP positive cells were observed using a Leica TCS SP8 laser scanning confocal microscope.

### RNAi vector construction and transformation

The backbone vector pGreenII 0000^37^ was generously offered by Singapore-Peking University Research Centre. The target sequence was synthesized based on a 198-bp fragment of *NsPDK*. A 1026-bp GUS sequence was chosen as the loop structure linking sense and antisense fragments. The endogenous promoter β-tubulin^29^ was used to drive the expression of both a bleomycin resistance gene and target sequence. The foreign terminator ribulose-1,5-bisphosphate carboxylase/oxygenase small subunit (RbcS1) from *Dunaliella tertiolecta* (GenBank accession AY530155) was used here. For nuclear transformation, plasmid was linearized with HpaI and transformed into *N. salina* via electroporation using Bio-Rad GenePulser Xcell apparatus (Bio-Rad, USA) at 12,000 V/cm, 50 µFD and 600 Ω. After electroporation, cells were incubated in 10 mL medium without antibiotics for 24 h, before they were transferred to f/2 plates. Zeocin (Invitrogen, USA) at the final concentration of 4 μg/ml (agar medium) and 1 μg/ml (liquid medium) was used for selecting transgenic cells.

### Molecular analysis of the transgenic microalgae by genotyping PCR, quantitative real time PCR (qRT-PCR)

Ten mL culture (about 400 billion cells) was harvested by centrifugation (5,000 rpm, 10 min at 4 °C). The cell pellet was washed twice, then suspended in 0.5 mL extraction buffer (2% SDS, 400 mM NaCl, 40 mM EDTA, 100 mM Tris-HCl, pH 8.0) at 65 °C for 30 min. The genomic DNA was extracted by addition of 0.5 mL phenol/chloroform/isoamyl alcohol mixture (25:24:1, Sigma-Aldrich, USA), followed by centrifugation (13,000 rpm, 30 min at 4 °C). The aqueous phase was mixed with equivalent volume isopropanol, inverted and centrifuged (12,000 rpm, 20 min at 4 °C). The DNA pellet was washed with 75% ethanol twice and dried for 2 h. PCR was performed with primers of GUS, the bleomycin resistance region, fragments crossing sense/GUS sequence and fragment crossing GUS/antisense sequence (Supplementary Table 1).

Quantitative real time PCR was performed in a BioRad CFX96 Real-Time System (BioRad, USA) in the presence of SYBR Premix (Thermo Scientific, USA). The profile was 50 °C for 2 min, then 95 °C for 10 min followed by 40 cycles of 95 °C for 15 s and 60 °C for 1 min, finally 65 °C for 5 s and 95 °C for 5 s. Data was collected at the end of each extension step. The relative quantitative of gene expressions in the treatment groups was analyzed by the 2^−ΔΔCt^ method, where the C_t_ was the cycle number at which the fluorescent signal statistically above the background. The actin amplification product was used as an internal standard.

### Determination of enzymatic activities

Twenty mL culture (about 400 billion cells) was harvested by centrifugation (12,000 rpm, 5 min at 4 °C). The cell pellet was washed twice, frozen with liquid nitrogen and pulverized with pestle and mortar. After extraction with extraction buffer (containing 100 mM KH_2_PO_4_/KOH (pH 7.5), 20% (v/v) glycerol, 1 mM benzamidine·HCl and 1 mM DTT) and centrifugation (12,000 rpm, 10 min at 4 °C), the supernatant was collected as a crude extract for enzymatic reactions. PDK activity was assayed with PDK Activity Elisa Kit (R&D system, USA) following the manufacturer’s instructions. PDH Activity Assay Kit (Sigma-Aldrich, USA) was used for determination of PDH activity, with pyruvate as substrate and NADH standard for quantification. One unit (U) of enzyme activity was defined as that amount of enzyme catalyzing the formation of 1 μmol of each enzymatic reaction product per min under the above-mentioned conditions. Soluble protein was measured with BCA Protein Assay Kit (Cwbio Biotechnology, China).

### Determination of cell concentration, chlorophyll and photosynthetic parameter

Cell number was calculated by counting under microscope using a hemocytometer.

Chlorophyll a in fresh algal cells (5 mL culture, centrifugation 12,000 rpm for 10 min) was extracted with 5 mL methanol for 5 h at 4 °C. By measuring the optical density respectively at 665 nm, 652 nm, 480 nm and 750 nm with a spectrophotometer U-3900H (Hitachi, Japan), the concentration was calculated with the following equation, and absorbances at 652 nm, 665 nm and 480 nm were corrected by subtracting absorbency at 750 nm (Ritchie, 2006).

[Chlorophyll a] mg/L = 16.5169 × A665−8.0962 × A652

Fv/Fm (the variable/maximum fluorescence ratio) indicates the maximum photochemical quantum yield of PSII reaction centers, reflecting the photosynthetic light energy conversion efficiency. The cells were kept in the dark for 15 min, then exposed to a saturating light pulse while the chlorophyll fluorescence intensities were measured with a OS5p chlorophyll fluorimeter (Opti-Sciences, US) following the manufacture’s recommendations.

### Determination of protein and carbohydrate content

For protein determination, 10 mg lyophilized algal biomass was hydrolyzed in 200 μL 1 M sodium hydroxide (NaOH) and then incubated in a water bath at 80 °C for 10 min. Then 1.8 mL H_2_O was added to the hydrolysate to bring the volume to 2 mL. The mixture was centrifuged at 12,000 rpm for 30 min and the supernatant was transferred to a new tube. This extraction procedure was repeated twice, and all the resulting supernatants were pooled together. Then the protein concentration was measured by the BCA Protein assay kit (Cwbio Biotechnology, China) with bovine serum albumin as the standard. For carbohydrate determination, 10 mg lyophilized algal biomass was incubated with 0.5 mL acetic acid at 80 °C for 20 min before 10 mL acetone was added, followed by centrifugation at 3500 g for 10 min. The pellet was resuspended in 2.5 mL 4 M trifluoroacetic acid and then boiled for 4 h to release reducing sugars. The suspension was cooled and centrifuged at 10,000 rpm for 5 minutes, and then 20 μL supernatant was mixed with 900 μL sulfuric acid:H_2_O:phenol (300:150:3, v/v/w) and boiled for 20 min prior to reading the optical density at 490 nm. Glucose was used to establish the standard curve for the quantification of total carbohydrate content.

### Total lipid extraction, TAG and fatty acid analysis

Total lipids were extracted from lyophilized algal cells in a chloroform-methanol-water (4:2:1.5, by vol) system^38^. The chloroform layer was evaporated under nitrogen gas and dried at 60 °C for 2 h in a vacuum oven. The lipids were then weighed for quantification. Total fatty acids were obtained via transesterification of total lipids and quantified by GC-MS.

TAG was separated on a TLC plate coated with silica gel 60 (Merck, USA) using a mixture of hexane/diethyl ether/acetic acid (70:30:1, by vol) as the mobile phase, while polar lipids were separated on a TLC plate using a mixture of chloroform/methanol/acetic acid/water (25/4/0.7/0.3, by vol) as the mobile phase. For quantification, lipids on TLC plate were visualized with iodine vapor, recovered, transesterified and analyzed by GC-MS.

Lipid transesterification was performed in 1% (by vol) sulfuric acid in methanol at 85 °C water bath for 2.5 h. Heptadecanoic acid (C17:0, Sigma-Aldrich, USA) was used as the internal standard. After extracting with hexane, the fatty acid methyl esters (FAMEs) were analyzed by using a GC-MS-QP 2010 SE (Electron Ionization type) gas chromatograph-mass spectrometer (Shimadzu, Japan) and a Stabilwax-DA capillary column (30 m × 0.25 mm × 0.25 μm) (Shimadzu, Japan). Helium was used as the carrier gas. The injection temperature, ion temperature and interface temperature were set at 250 °C, 200 °C and 260 °C, respectively. The initial column temperature was set at 150 °C. The column temperature subsequently rose to 200 °C at 10 °C/min and then to 250 °C at 15 °C/min, followed by a hold at 250 °C for 3 min. FAMEs were identified using the NIST 11 mass spectral library (NIST/EPA/NIH mass spectral library, 2011 edition).

### Statistical analysis

All data were obtained by using at least three biological samples to ensure the reproducibility of the results. The statistical significance of the results was evaluated by independent samples t-test and one-way ANOVA Turkey’s HSD tests (*p* < 0.05) using the SPSS version 16.

### Data availability statement

All data generated or analyzed during this study are included in this published article and its Supplementary Information files.

## Electronic supplementary material

supplementary information
