# Supplementary material for: RNAi-mediated silencing of a pyruvate dehydrogenase kinase enhances triacylglycerol biosynthesis in the oleaginous marine alga Nannochloropsis salina
Source: Sci Rep. 2017 Sep 13;7:11485. doi: 10.1038/s41598-017-11932-4 (PMC5597597; doi:10.1038/s41598-017-11932-4)
Supplement: Supplementary file 1 — supplementary information [file 41598_2017_11932_MOESM1_ESM.pdf]

RNAi-mediated silencing of a pyruvate dehydrogenase kinase enhances triacylglycerol biosynthesis in the oleaginous marine alga *Nannochloropsis salina*

Xiaonian Ma [1, 2], Lina Yao [3], Bo Yang [1], Yuan Kun Lee [3], Feng Chen [1, 2 *] and Jin Liu [1, 2, *]

[1] *Institute for Food and Bioresource Engineering, College of Engineering, Peking University, Beijing 100871, China*

[2] *BIC-ESAT, College of Engineering, Peking University, Beijing 100871, China*

[3] *Department of Microbiology and Immunology, Yong Loo Lin School of Medicine, National University of Singapore, 117545, Singapore*

⋆ Corresponding authors. E-mail: sfchencoe@pku.edu.cn (F.C.); gjinliu@pku.edu.cn (J.L.)

Supplementary Table 1 Primer pairs used in the present study.

Gene name	Primer sequence	Use	GenBank accession no.
PDK	PDKf: ATGATAGCACGTCGTGTACA<br>PDKr: TTAGACCAATGGCTCCTTGC	Full-length amplification	XM_005855411
	PDK_KpnI_f: GGGGTACCATGATAGCACGTCGTGTAC<br>PDK_KpnI_r: GGGGTACCGACCAATGGCTCCTTGCA	PDK-GFP fusion construction	
	kd1_anti_f: AAAACTGCAGACGCGTCAGCACCTTCACGA<br>kd1_anti_r: CTAGACTAGTGCGACCTTCTTGCACCGAGA	Sense sequence amplification	
	kd1_sense_f: CCCATCGATGCGACCTTCTTGCACCGAGA<br>kd1_sense_r: CCGGAATTCACGCGTCAGCACCTTCACGA	Antisense sequence amplification	
	PDK_qf: CTCGACGAACTCCATCTGAA<br>PDK_qr: CCGTCTCGATCACATCTTGA	Real time PCR	

Supplementary Table 1 Primer pairs used in the present study. (Continued)

eGFP	eGFP_ KpnI_f: GGGGTACCGGACCTAGGGGAGGAGGAGGAGGAATGGTGAGCA AGGGCGAG<br>eGFP_XbaI_r: GCTCTAGATTACTTGTACAGCTCGTCCATGC	PDK-GFP fusion construction	
β-tubulin	TUBf: CGGGGTACCACTGCGCATGGATTGACCGA<br>TUBr: CCGCTCGAGTGCTTCACAAAAAAGACAGCTTCTTGAT	Promoter	
RbcS1	RbcS1_f: GCTGTTAGCACAGCACTTGC<br>RbcS1_r: CCATCCTTTCTGTACTGCTT	Terminator	AY530155
GUS	GUS_f: GATATCTACCCGCTTCGCGT<br>GUS_r: CACCGAAGTTCATGCCAGTC	Genomic PCR detection	
Bleomycin	Bleo_f: TCGAGTTCTGGACCGACCGGCT<br>Bleo_r: TCCTGCTCCTCGGCCACGAAGT	Genomic PCR detection	
Sense-GUS	Sense-GUS_f: ACCTTCTTGCACCGAGAGCT<br>Sense-GUS_r: ACGGCGCTGACGCGATCAAA	Genomic PCR detection	

Supplementary Table 1 Primer pairs used in the present study. (Continued)

GUS-antisense	GUS-antisense_f: GCGTGGCAAAGGATTCGATAACG<br>GUS-antisense-r: GACCTTCTTGCACCGAGAGC	Genomic PCR detection	
Actin	Actin_f: ATTTCGTGCGCCGGAGG<br>Actin_r: GTCGCACTTCATGATCGTCTG	Real time PCR	XM_005852283
PDHC_E1	PDHCe1_f: TTGGTCAATCGCAGTATGGT<br>PDHCe1_r: CACCTCCTCAAGCTGTTTCA	Real time PCR	AZIL01000171.1
PDHC_E2	PDHCe2_f: AGCCACTGAAACAACACCAG<br>PDHCe2_r: AGCCACTGAAACAACACCAG	Real time PCR	XM_005854674.<br>1
ICDH	ICDH_f: CTCACTGGGCCTTATGACCT<br>ICDH_r: GGCTTGCCTTTCTGGTACTC	Real time PCR	AZIL01000117
CS	CS_f: ATGGAAACAGCCTTCTTGCT<br>CS_r: CCGTCTGATGATGGTAAACG	Real time PCR	AZIL01001543
ME	ME_f: ACTCCAAGGGCTTGATTGTC<br>ME_r: AATTCGTCCGACCTCTATGC	Real time PCR	AZIL01000703.1

Supplementary Table 1 Primer pairs used in the present study. (Continued)

G6PDH	G6PDH_f: CGAATTACGAGACCAGCTCA<br>G6PDH_r: CCCGACTTGTACATGCACTT	Real time PCR	AZIL01000829.1
PDC	PDC_f: AACAACCCTGACACAGTGGA<br>PDC_r: CGCACCTTCACACCTATGAC	Real time PCR	XM_005854810
ALDH	ALDH_f: CGAAGCAGAGGCAGACATAA<br>ALDH_r: TCCATTCATGGAGGTGCTTA	Real time PCR	AZIL01001205
ACS	ACS_f: CAGCCGCTTGTTGAGTGTAT<br>ACS_r: CCCAGCATATTCGAGCCTAT	Real time PCR	JU980159.1

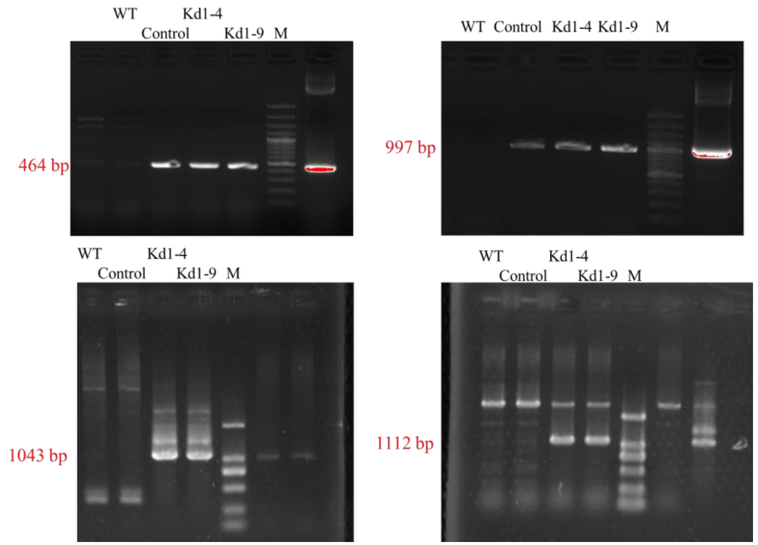

Supplementary Figure 1. Detection of bleomycin resistant fragment (464 bp), GUS region (997 bp), fragment crossing sense and GUS sequence (1043 bp), fragment crossing GUS and antisense sequence (1112 bp) from transgenic *N. salina* (shown in full-length gels).

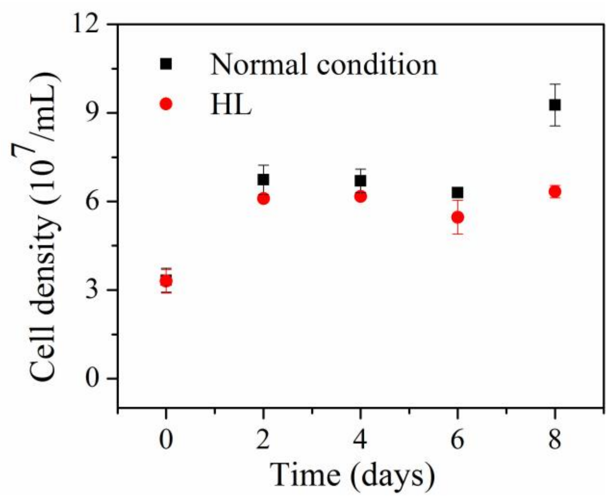

Supplementary Figure 2. The time curve of cell numbers in *N. salina* under normal conditions (50 μmol/m^2/s) and high light conditions (250 μmol/m^2/s).

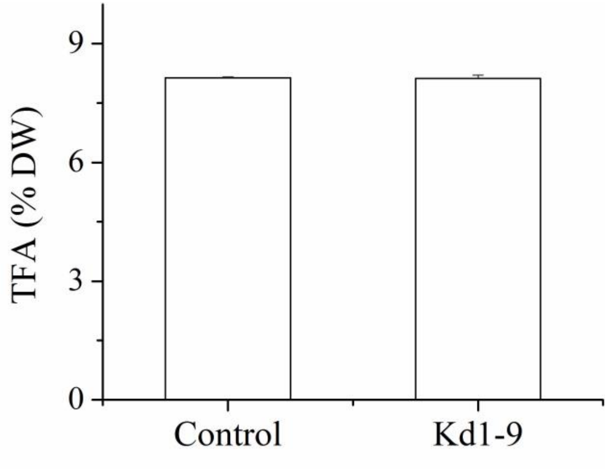

Supplementary Figure 3. Total fatty acid content in control and Kd1-9 cells on day 0 before transferred into HL conditions. DW, dry weight.

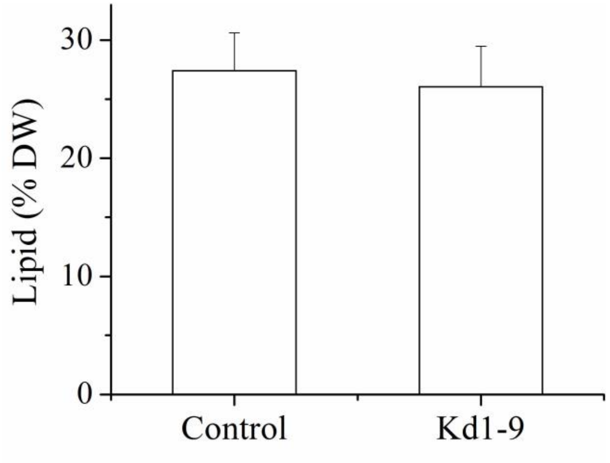

Supplementary Figure 4. Total lipid content in control and Kd1-9 cells on day 2 under nitrogen deficiency conditions. DW, dry weight.

Supplementary Data 1 Coding sequence of putative mitochondrial pyruvate dehydrogenase kinase from *Nannochloropsis salina*

ATGATAGCACGTCGTGTACAGTTTTTGAAAACAAATTTATATCAGTATGCGTCAAAGCGTAGATTTGCACAGATCGCCACGTCTTCTGCCCTTTTTCCCC
ATCAGTCGCATCTCCAGCAGCACCAGCAACACGGACAAGTAGGCGCTGCAGGAAGCTCCACGCCTTCTTTTTTGCAACAGCAGACTCTTAAAGCGGAT
AAAATACCTACGCCTACGAAAATAGCAGGTGCATCCGTGCCTTGGTCAGATTCAGCTCACAGCGTAAGCGAGTCGAAATCGGAAATCCTCCGAGACGA
AGAAGCGACTATACTTACATATGCGAAGAAACGGTCGGAACGGGTGTCTCTGCGCAGTCTGGCGGAAGTGGGCTTGGGAACGCGACCTTGGTTGTCG
AGTCTAAGAGATGCCATGTCCCTGGAAAATCCCCTCTGTTCCTTGAACGTGCGTCTCTGCAGGGTGTCTGACCCCCACTCCCCGGCCCAGAAGGGGCT
TCTGCGCATGGCGACCTTCTTGCACCGAGAGCTCCCGATTCGCTTCGCACGCGGCATCACTTTCATCGACAAATTAGATTCAAGTCGACAGGCGCCCA
GTTTGCGTGTGGTTCGAGAGTGGTACAGGGAATCCTTTCGAGACGTGGTGTCAAGCCCGTGCCCCGTCACCGACGGCTGCGAGGAGAGCTTCGTGAA
GGTGCTGACGCGTGTCCGGGACAGGCACGCGGACGAACTCTTGCTGGTTGCGCGAGGGGTCTTTGAACTGCGAGCCAAGCTAGGTATGGACGGTTTG
GACGGGAGGGGCGGTCGGGAAGCTCTCCACGCGCAACTCGACGAACTCCATCTGAAGCGCATAGCCCTCCGCATCCTCGTGGGTCATTACCTGGCTCT
GCACCAGCCCCCTCGCCCCAATTACGTGGGGATAATCTGCACGCGCACAAAACTTCAAGATGTGATCGAGACGGCGGCTGCGGACGCACGCTGGATTT
GCAAGCAACGCTTCGACGGTTGCGCCCCGCGAGTGGAGGTGATTGGGGGGGAGGGTATGGTGATGGCCTGCATTCCGGAAAGCTTATACTATCTGTCG
ATGGAACTCATCAAAAATTCATTGCGGGCAGTAGCAGAGCGCTACAGTGAGGCTTTGTTTCGTGACGGATCAATGGCACATGGCGCGGGCGGTGACGA
AGGGTGTGTGCCCTCGATCAAAGTCATTYTCAGCCGAGAGTACAGTTTGACAGAAGGGCAACAGGTGGTGATCGAGGTGCGGGACGAAGGGGGGGG
CATTCCGCCGGAGGATCTAGGTAAGGTTTTCTGTTACCTTTTTTCCACTGCGGCTGATGCGGATGTGCAGCAACTCGTTATGGACCGTCACGCCTCGGG
GCTTGGGGGGGAAAGGAGTAAGCACAACAGAGGGAACTCAAAGAAAGTGCCTCTCGCAGGTCTGGGGTATGGTTTGGGCATTGCCAAGTCATATGCT
TTGTATTTTGGGGGGGAGTTGGAAATCAAAAATCGGCCGGGAGATGGCTGCTCGGTGTTTGTGACTTTGAGCCGGCTTGGTGAGTGCAAGGAGCCATT
GGTCTAA
